# Review of Vaginitis

**DOI:** 10.1155/S1064744993000341

**Published:** 1993

**Authors:** Sebastian Faro

**Affiliations:** Department of Gynecology and Obstetrics University of Kansas School of Medicine 3901 Rainbow Boulevard Kansas City KS 66160-7316 USA

## Abstract

Adisruption of the dynamic equilibrium of the healthy vagina may have significant sequelae, leading to chronic or serious conditions. Therefore, all cases of vaginitis should be accurately diagnosed and appropriately treated.


Infectious Diseases in Obstetrics and Gynecology 1:153–161 (1993)
© 1993 Wiley-Liss, Inc.

### Review of Vaginitis

**Sebastian Faro**

*Department of Gynecology and Obstetrics, University of Kansas School of Medicine, Kansas City, KS*

ABSTRACT

**A disruption of the dynamic equilibrium of the healthy vagina may have significant sequelae, leading to chronic or serious conditions. Therefore, all cases of vaginitis should be accurately diagnosed and appropriately treated.** © 1993 Wiley-Liss, Inc.

KEY WORDS
Vaginal microflora, vaginal discharge, vaginal pathogens

Vaginitis is probably the most common reason for patients seeking a physician's care for problems related to the reproductive tract. Vaginitis is estimated to be responsible for 10% of such office visits.[1] Thomason et al.[2] reported that bacterial vaginosis accounted for 31.1% of the cases seen in their ambulatory practice. In addition, they found that 23.9% of these cases were due to yeast, 12.9% due to human papillomavrius (HPV), 8.9% to unknown causes, 6.1% to bacterial vaginitis, and 2.7% to atrophic vaginitis.

Although vaginitis is sometimes considered to be nothing more than a nuisance, it has been implicated in significant sequelae in perinatal, postpartum, and postgynecologic surgery patients. There is some disagreement on a direct cause-and-effect relationship between the presence of vaginitis and subsequent morbidity in the obstetric and gynecologic patient, but it stands to reason that a vaginal microflora dominated by pathogenic bacteria is potentially a threat to the patient's well being. An abnormal microflora becomes of even greater concern if conditions are present that increase the patient's vulnerability.

Vaginitis may be due to infectious or noninfectious causes. Infectious causes present the physican with a spectrum of etiologic agents including bacteria, fungi, protozoa, and parasites. At times identifying the etiologic agent is simple, while at other times it is very difficult. Nevertheless, the most important factor leading to successful treatment is establishing the correct diagnosis.

### MICROBIOLOGY

Understanding the various types of microbial vaginitis requires an understanding of the microbiology of the healthy vagina. The endogenous microflora constituting a major part of the vaginal ecosystem consists predominantly of commensal bacteria. Potentially pathogenic bacteria, although in lower concentrations, are also present. The commensal organisms exist in a synergistic state with one another, but are antagonistic toward the potentially pathogenic bacteria. When environmental conditions are appropriate, the commensal bacteria suppress the growth of pathogens.

The dominant bacteria in the healthy vagina are *Lactobacillus*, *Corynebacterium*, and diptheroids. The pathogenic bacteria consist of gram-positive and gram-negative aerobes and facultative as well as obligate anaerobic bacteria. There are many species of lactobacilli: *L. acidophilus*, *L. fermentum*, *L. brevis*, *L. casei*, *L. leichmani*, *L. salivarious*, *L. lactis*, and *L. cellobiosis*.[3] Lactobacilli, especially *L. acidophilus*, play a central role in maintaining the proper environment by producing lactic acid and

**Address correspondence/reprint requests to Dr. Sebastian Faro, GYN/OB University of Kansas School of Medicine, 3901 Rainbow Boulevard, Kansas City, KS 66160-7316.**

Received October 5, 1993

Accepted October 19, 1993

Review Article hydrogen perioxide ($H_2O_2$). The pH range of the healthy vagina is 3.8–4.5, and the proper hydrogen ion concentration is maintained through the production of lactic acid. The pathogenic bacteria, especially anaerobic bacteria, tend not to grow well at the healthy pH range. Hydrogen peroxide is toxic to anaerobic bacteria because they do not produce catalase and thus cannot convert hydrogen peroxide to water and oxygen. Hydrogen peroxide is a bacterial antagonist that functions directly on the bacterial cell or a peroxidase mediate enzymatic system.[4,5] Eschenbach et al.[6] demonstrated that 96% of women with a healthy vaginal flora have hydrogen peroxide-producing lactobacilli. On the other hand, they found that only 35% of women with a diagnosis of bacterial vaginosis had lactobacilli, of which only 11% produced hydrogen peroxide.

Lactobacilli also produce lactocins which are protein-inhibiting bacteriocins.[7] These inhibitors have a narrow spectrum of activity that includes the strains from which they were produced.[8] These bacteriocins are an effective means of controlling bacterial growth. They also maintain lactobacilli counts within a range that prevents them from exerting an adverse effect on the host. They are limited in their activity in that some are effective only against gram-positive bacteria and others only against gram-negative bacteria.[9] Data suggest that lactobacilli may inhibit bacterial adherence to epithelial cells,[10,11] which is a significant factor in view of the fact that the infectious potential of bacteria depends upon their ability to adhere to epithelial cells.

The maintenance of the vaginal microecosystem is dependent upon the dynamic interaction of bacteria and host factors. An important aspect of this equilibrium concerns the synergism and antagonism occurring in the endogenous microflora of the lower genital tract. Organisms introduced from the exogenous environment must overcome endogenous organisms in order to disrupt the equilibrium of this microecosystem. However, when *Neisseria gonorrhoeae* or *Chlamydia trachomatis* are introduced in the lower genital tract, the endogenous factors become significant because these organisms do not reside in the vagina and infect the more vulnerable columnar epithelium of the endocervix rather than squamous epithelial cells.

### PATIENT EVALUATION

The initial step in the evaluation of the patient with a complaint of vaginitis is to obtain a detailed history. This step is critical in the necessary consideration of all possible factors that may impinge upon the vaginal microecosystem and disrupt the equilibrium of the healthy state. This delicate equilibrium can easily be disturbed by endogenous or exogenous factors.

The physician should elicit information regarding the patient's hygienic practices, change in soaps, douching agents and frequency, and type, dosage, and frequency of any medications. Antibiotics have been shown to alter the endogenous flora. Cephalosporins, e.g., in a single dose, have been shown to cause an increase in colonization by enterococci.[12]

Determining how sexually active the patient is is significant because an increase in prevalence of bacterial vaginosis has been associated with an increased frequency of sexual intercourse. Also important to determine is whether the patient's pattern of sexual behavior or practices provide an opportunity for vaginal colonization by oral or fecal flora. These bacteria may present a synergistic opportunity when placed in the same environment with endogenous pathogens, thereby becoming the dominant flora.

The patient should be given the opportunity to describe her symptoms in detail, characterize the discharge, and specifically point out the anatomic site of her symptoms. Often times, if the patient does not specifically describe and locate her symptoms, the physician and the patient mistakenly presume that she has a vaginitis. She may believe that she has a vaginal infection yet identify the location of her discomfort (usually burning, itching, or pain) at the opening of the vagina or she may reveal that she has dyspareunia on insertion of the penis.

Other important information is when the condition first occurred and specific treatments that have been used. Knowing the duration of the disease and treatments already administered will help the physician to explain the complexity of the problem to the patient. At the same time, the characteristics of a healthy vagina should be explained to the patient (Table 1).

Typically, the patient with a healthy vagina has a slate-gray to white discharge with no odor. The estrogenized vagina normally is pink-white with

TABLE 1. Characteristics of a healthy vagina

| | |
|---|---|
| pH | Range 3.8–4.5 |
| Discharge | Liquid to creamy-pasty |
| Color | Slate-gray to white |
| Odor | None |
| Background | Free-floating bacteria, mostly bacilli |
| WBCs | Rare |

rugae. The presence of petechial hemorrhages on the vaginal epithelium indicates an abnormal vaginal microecosystem. The presence of an odor emanating from the pelvic area may not originate in the vagina but may indicate poor hygiene, hidradenitis, or fecal soiling.

The vaginal discharge should be examined microscopically by swabbing the lateral vaginal wall with a cotton-tipped swab. The swab is then inserted into a tube containing approximately 2 ml of saline and swirled to dilute the vaginal discharge. A drop or two of the diluted discharge is placed on a glass slide and a coverslip is placed over the specimen. The specimen should be examined at ×40 magnification. The healthy vaginal discharge will contain squamous epithelial cells whose cytoplasmic and nuclear membranes are easily seen. Bacteria will be individually free floating in the vaginal fluid and will be largely bacillary morphotypes of equal size (lactobacilli). There may be a rare white blood cell (WBC).

The pelvic examination should begin with a close inspection of the vulva to determine the presence of lesions, e.g., pyodermas, folliculitis, carbuncles, hidradenitis, raised white pyriform growths, raised umbilicate lesions, ulcers, erythema, or excoriations. If there is any suggestion that the patient may have HPV, then later, after the vagina has been evaluated, the vulva should be painted with 5% acetic acid and examined under magnification. The Bartholin's and Skene's glands should be palpated and an attempt made to express the contents of these glands. If a purulent discharge is present, it should be Gram stained and cultures should be taken for aerobic, facultative, and obligate anaerobes as well as *C. trachomatis*. A vaginal speculum should be inserted with care to avoid traumatizing the cervix, which is likely to result in bleeding if the cervix is inflamed. The pH of the vagina should be ascertained by applying pH paper to the lateral vaginal wall. Both semen and spermicides will cause the pH to be more alkaline, so it is prudent to determine if the patient has had sexual intercourse in the preceding 24 hr and if a spermicidal gel has been used. By performing a microscopic examination of a diluted specimen of the vaginal discharge, the physician should be able to determine whether the vaginal microecosystem is healthy or not (Table 2).

After performing the pelvic examination, the physician should be in a position to construct a differential diagnosis (Table 3). Following the microscopic examination of the vaginal discharge, a definitive diagnosis will be possible in most cases. On occasion, the examination will not reveal the etiology of the patient's problem and specialized testing such as cultures for *Candida* or *Trichomonas* may be necessary.

In 75–80% of the cases, *T. vaginalis* can be diagnosed by microscopic examination of the vaginal discharge. The organism is easily identified by the motile anterior flagella and characteristic movement on microscopic examination of an unstained preparation of diluted vaginal discharge. The vaginal discharge in itself does not possess any specific characteristics that aid the physician in establishing a diagnosis without further analysis. A frothy, dirty-gray discharge can be seen with a trichomonas infection as well with bacterial vaginosis or *Gardnerella vaginalis*. The classic "strawberry cervix" is seen in only approximately 25% of cases. If there is strong suspicion of trichomoniasis but no identifiable trichomonads, a portion of the vaginal discharge can be placed into a commercial system for trichomonas culture to allow the trichomonads to grow. This culture should be examined daily for the presence of trichomonads. Trichomonads can also be identified on a Pap smear. Typically, the bacterial flora will have shifted from lactobacillus-dominated flora to a polymicrobial one toward anaerobes.

The presence of trichomonads should alert the physician that the patient is at risk for other sexually transmitted organisms and that further testing may be indicated. A patient with trichomoniasis may also have a concomitant bacterial vaginosis. In such a patient, treatment with metronidazole will often be effective in correcting both infections. Occasionally, a patient may have a concomitant candidi-

TABLE 2. Characteristics of vaginal discharge

| Characteristic | Healthy vagina | Bacterial vaginosis | *Gardnerella vaginalis* | *Trichomonas vaginalis* | *Candida* vaginitis | Atrophic vaginitis |
|---|---|---|---|---|---|---|
| pH | 3.8–4.5 | >4.5 | >4.5 | >4.5 | <5 | >3.8 |
| Color | Gray-white | Gray | Gray | Gray | White | White-yellow |
| Odor | None | Fishy | Fishy | Foul | None | None |
| Whiff test | Negative | Positive | Positive | Positive | Negative | Negative |
| Clue cells | Negative | Positive | Positive | Negative | Negative | Negative |
| Background | Bacilli | Mixed | Clumps | Mixed | Bacilli | Rare |
| WBCs | Rare | +− | Rare | Yes | Yes | Yes |
| Bacteria | Bacilli | Mixed | Bacilli | Mixed | Bacilli | Rare |

TABLE 3. Differential diagnosis for vulvovaginitis due to infection

| Vulva | Vagina |
|---|---|
| *Candida* | Bacterial vaginosis |
| Folliculitis | *Gardnerella vaginalis* |
| Carbuncles | *Streptococcus agalactiae* |
| Hidradenitis | Herpes simplex |
| Herpes simplex | Human papillomavirus |
| Human papillomavirus | *Trichomonas vaginalis* |

asis that can usually be detected on a wet-mount preparation. If difficulty is encountered in identifying pseudohyphae, yeast, or budding yeast forms, a drop or two of the vaginal discharge can be mixed with a drop or two of 10% potassium hydroxide (KOH). The chitinous cell walls of the fungi, which are resistant to strong alkaline solutions, will not dissolve as the squamous epithelial and bacterial cells will.

A microscopic examination of the patient's vaginal discharge may not reveal the presence of trichomonads. However, if it contains a significant number of WBCs and numerous bacteria of various morphotypes, but no clue cells or pathogens, a culture of the discharge specimen should be taken for *T. vaginalis*. Consideration should also be given to the possibility of an HPV infection.

Some debate concerns the issue of whether bacterial vaginosis is different from *G. vaginalis* vaginitis or whether the latter is just a marker organism for bacterial vaginosis. Additional so-called marker organisms are *Mycoplasma* and *Mobiluncus*. A review of the original works of Gardner and Dukes[13] makes it apparent that these two conditions may not be the same or may represent a spectrum of the same condition. These authors described clue cells as the presence of gram-negative bacteria (*G. vaginalis*) adhering to squamous epithelial cells. Their photomicrographs show that the bacteria in the vaginal fluid not attached to squamous epithelial cells are not individually free floating but clumped or in aggregates with a characteristic lack of WBCs. *G. vaginalis* is a constant factor in the bacteriological assessment of bacterial vaginosis, as it is usually present in significant numbers. Perhaps the two conditions should be viewed as a spectrum of bacterial vaginitis. In the initial phase when the environment is in the stage of being disrupted, a decline in lactobacilli occurs resulting in a shift in hydrogen ion concentration and allowing the growth of *G. vaginalis*. The continued growth of *G. vaginalis* results in further decline in the hydrogen ion concentration and growth of the anaerobes. This continual decline in hydrogen ion concentration favors the growth of anaerobes, eventually leading to the typical microbiological flora of bacterial vaginosis. This hypothesis has some support in the clinical setting by observing patients who have been repeatedly treated with metronidazole but have failed to be cured. Microbiological analysis of the vaginal flora of these patients makes it possible to divide them into 2 groups: 1) those with nondescript flora dominated by *Staphylococcus epidermidis, Enterococcus faecalis,* or *Escherichia coli* and 2) those with flora dominated by *G. vaginalis* and a conspicuous absence of anaerobes.[14,15] Therefore, it would seem logical that patients in the early development of a derangement of the vaginal microflora dominated by *G. vaginalis*, which is typically resistant to metronidazole, would not be cured by this drug.

Individuals presenting with a clinical picture of bacterial vaginosis or *G. vaginalis* should not have their vaginal specimens sent for culture if the laboratory is not equipped to perform a thorough workup. The treatment options are limited and chosen empirically. Patients with repeated or chronic vaginitis should be thoroughly evaluated with specimens obtained for the culture of fungi, trichomonads, *Streptococcus agalactiae*, and, if laboratory capabilities exist, aerobic, facultative, and obligate anaerobes. Again, if the laboratory is not equipped to carry out these studies, obtaining these specimens will be a waste of money. If vaginal specimens are sent to the laboratory, they must be transported in anaerobic vessels. Sending only aerobic specimens will usually result in reports of a single organism, e.g., *E. coli*. This manner of handling specimens usually leads to treatment with an inappropriate antibiotic and failure of the patient to respond. It is extremely important that, when a patient complains of recurrent vaginitis, the physician take time to examine the genital area thoroughly and specifically avoid administering antimicrobial agents that may have an adverse effect on the vaginal flora and result in continuation of the patient's symptoms.

Yeast cells are commonly seen in the patient's vaginal fluid, being isolated from approximately 40% of asymptomatic patients with a healthy vaginal microflora.[16] There has been much discussion regarding the development of more specific and sensitive tests for the identification of yeast forms in patients with complaints of vaginitis. Approximately 75% of cases can be diagnosed by microscopy and KOH. The rapid tests that have been developed are only about 81% effective. Typically, the patient with a yeast vaginitis will present with complaints of vulvovaginal itching. Inspection of the vulva may or may not reveal the presence of erythema and excoriation. The vaginal epithelium as well as the cervical epithelium are erythematous. The discharge is classically thick and pasty, often referred to as "cottage cheese-like," but it may be homogenous and liquid. Usually, no odor is present, but when present it typically has the aromatic odor of yeast. Microscopic examination of a diluted portion of the vaginal discharge may reveal the presence of elliptical yeast cells that refract light and tend to have a greenish tinge to the cytoplasm. The cells may be budding or have short germination tubes, which represent the beginning of the production of the characteristic pseudohyphae. When the pseudohyphae are quite long and branching, they can usually be found among clumps of squamous epithelial cells. In the event that these structures cannot be identified, an aliquot of vaginal discharge can be placed on a glass slide and 1 or 2 drops of 10% KOH can be added to it. Fungal and yeast cell walls are made of chitin and resistant to strong alkaline solutions. Therefore, all nonchitinous structures, e.g., bacteria and squamous cells, will dissolve in the alkaline solution. The remaining fungal cells are easy to identify. In recurrent cases or in cases in which the organisms are not visually identified, a culture is recommended. Such a culture can easily be accomplished by placing a specimen of the vaginal discharge on Sabouraud's or Nickerson's medium. In recalcitrant cases, the organism should be identified according to species. Antifungal sensitive studies may need to be performed in patients having tried without success all vaginal and oral preparations commonly used.

### TREATMENT

The first error in the management of vaginitis occurs when an antimicrobial agent, even if nothing more than a sulfa-based cream, is administered to the patient presenting with vaginitis. If the etiology of the vulvovaginitis has not been found, it is more appropriate to try to correct the pH if it is not within the acceptable range (3.8–4.5) than to prescribe an antimicrobial agent. Perhaps the patient should do no more than douche with vinegar water. Again, it is critical to establish the precise location of the symptoms.

Patients whose complaints are truly localized to the inferior aspect of the vestibule, specifically the posterior fourchette, should be first managed by attempting to establish whether the problem is chronic inflammation, vestibulitis, or HPV, or a combination of these conditions. The diagnosis can be established by performing colposcopy and biopsy of the affected area. Patients with chronic inflammation are best managed by the local application of hydrocortisone, 1% or 2.5% lotion, cream, or ointment. Although this therapy can be instituted prior to performing a colposcopically directed biopsy, it can cause inconvenience and unnecessary cost to the patient if a return visit is necessary to continue the evaluation. A patient with vestibulitis requires histologic confirmation of her condition. The tissue typically reveals replacement of the gland acini (vestibular glands) with stratified squamous epithelium and inflammatory cells. Treatment involves surgically excising the vulvar tissue containing the vestibular gland.[17–21] In some instances, the patient may have either of these diagnoses associated with the presence of an HPV infection, in which case laser ablation may be of value. Prior to instituting any form of treatment, however, the presence of other possible causes of vulvar discomfort such as herpes or lichen planus must be ruled out.

The treatment of *T. vaginalis* is best accomplished with oral metronidazole. Intravaginal metronidazole should not be used alone as it is not effective in eradicating trichomonads found, in the bladder, upper genital tract, or Skene's glands. Therefore, oral preparations should be used. The only drug approved in the United States is metronidazole. Trinidazole, which can be obtained in other countries, has a longer half-life. A single oral 2-g dose of metronidazole has been shown to be 90% effective. If the sexual partner is treated at the same time, the effectiveness is 95%.[22,23] This drug can also be administered in dosages of 250 mg 3 times daily or 500 mg twice daily for 7 days. The most common side effects are metallic taste, nausea, and vomiting. Patients should be strongly advised not to consume alcoholic-containing liquids while taking this medication and should refrain from them for 24 hr after the final dose. The combination of metronidazole and alcohol produces a disulfiram-like reaction with nausea and abdominal cramping.[24]

There is some concern over the carcinogenic potential of metronidazole, which is related to the detection of mutagens in the urine of patients taking this drug.[25] These weak carcinogens have been reported to cause alterations in DNA. Thus far, no large studies have addressed this concern, but two small studies have shown no documented relationship between metronidazole use and cancer.[26,27] In cigarette smokers who have used metronidazole, a 10-fold increase in lung cancer has been reported.[26]

Metronidazole is relatively contraindicated in the pregnant patient. The role of trichomoniasis in adverse pregnancy is undergoing intensive investigation. This antibiotic does not appear to have teratogenic effects. However, in two relatively small studies, congenital anomalies were found in infants born to women who ingested metronidazole in the first trimester.[26,28] Because of its possible teratogenic and carcinogenic effects, metronidazole should not be given in the first trimester and should be used cautiously in the second and third trimesters.[28] Treatment should be reserved for only those individuals who are truly symptomatic. An alternative to metronidazole is clotrimazole. Clotrimazole can inhibit the growth of *T. vaginalis*,[29] but it will not eradicate the trichomoniasis and the infection will recur.

Women who are breast feeding must be advised that metronidazole achieves detectable levels in breast milk.[30] For this reason, breast-feeding women who must be treated for symptomatic infection should pump their breasts and discard the milk for at least the first 24 hr after discontinuing metronidazole. An appropriate course of management for the breast-feeding patient being treated for trichomoniasis is to collect an adequate supply of breast milk before instituting metronidazole therapy; discontinue breast feeding but pump the breasts; take a single 2-g dose; and resume breast feeding 24 hr after completion of therapy.

Any patient who fails routine treatment regimens should be questioned to determine the degree of her compliance in taking the medication as directed. In addition, any possibility of reinfection should be pursued. Reinfection can occur if her partner did not take the medication or she acquired a new partner. Although *T. vaginalis* has remained relatively sensitive to metronidazole, resistant strains have been reported.[31] Sensitivity testing to metronidazole is complicated by the possibility that the test was not conducted in a truly anaerobic environment, as resistance has not been demonstrated in a truly anaerobic environment.[31,32] Oxygen has been shown to affect the efficacy of metronidazole.[33] Therefore, since the vaginal ecosystem is complex, the oxygen reduction potential of the vaginal ecosystem will undoubtedly have a significant effect on the effectiveness of the drug in the treatment of trichomoniasis. Resistant or chronic infection can be treated by increasing the dose of metronidazole. One regimen is the administration of 2–3 g orally daily for 14 days with the concomitant use of 500 or 1,000 mg vaginal suppositories daily or intravaginal metronidazole 2–3 times a day for 14 days. Utilization of high doses of metronidazole will, in all likelihood, cause significant nausea. One investigator reported that strains of *T. vaginalis* with an aerobic MIC >400 mg required a total of 40 g of metronidazole over a 14-day treatment course.[32,33] Some patients have also been treated with intravenously administered metronidazole, 3–4 g/day.[34] However, it must be pointed out that the administration of metronidazole at or above 4 g involves significant potential for the induction of seizures and disabling peripheral neuropathy.

Treatment of yeast vaginitis can be carried out with a number of antimycotic agents that can be placed in the vagina either in suppository form or as a cream. These agents are primarily azole derivatives: butoconazole, clotrimazole, miconazole, and terconazole. The duration of therapy ranges from 3 to 7 days. *Candida albicans* is the most common cause of yeast vaginitis and remains sensitive to the azoles. However, *C. glabrata* and *C. tropicalis* both tend to be resistant to most azoles, with terconazole having good activity against these species.[35,36] Another effective treatment is nystatin (polyene), but it should be considered a second-line agent because it has not produced the success that the azoles have achieved. Another polyene commonly used in systemic fungal infections is amphotericin B, which is also supplied as a 3% cream or ointment. It is typically used for cutaneous or mucocutaneous infection and has not been approved for vaginitis. Although amphotericin B can be utilized for chronic or recurrent infection, it should be used with caution as no data are currently available on its systemic absorption.

Other forms of therapy that have been used with success are boric acid suppositories, 600 mg, placed intravaginally twice daily for 10–14 days. Boron has not been detected in the bloodstream following intravaginal usage, but it should not be used in pregnancy because no studies have been conducted on pregnant patients.[37] Gentian violet used as tablets, paint, or tampons has proven to be effective, but it is somewhat difficult to use because it stains the clothing and skin.

Oral therapy has been shown to be effective with ketoconazole, 400 mg/day for 14 days, followed by a maintenance dose of 100 mg/day for 6–12 months. The patient's liver function must be monitored because liver toxicity in patients using ketoconazole for long periods is possible. Fluconazole can also be given orally, but there is no indication of it in the treatment of vaginal candidiasis. The dosage has not been established, but I have had success with an initial dose of 200 mg followed by 100 mg daily for 7 days.

Patients with recurrent or chronic infection should be evaluated for any underlying systemic illness such as diabetes or immunodeficiency. Again, a thorough history is important, especially with regard to any antimicrobial therapy that the patient may be on. The patient's hygiene and sexual behavioral patterns should be reviewed. In addition, the possibility of gastrointestinal colonization should be considered as significant colonization of the gastrointestinal tract and oral cavity has been reported in patients with recurrent or chronic infection.[37] Thus far, the available data do not indicate that oral nystatin is beneficial in these patients over a long period. The data do indicate a benefit over the short term.[38] The possibility that the organism may colonize the sexual partner who, in turn, reintroduces the organism to the vagina of the patient with each subsequent intercourse should also be considered. Studies have demonstrated that approximately 20% of the male sexual partners of women with candidiasis are colonized.[39,40] For this reason, it is probably beneficial to treat the male partner with an agent such as fluconazole while treating the patient. I prefer an oral regimen over a topical one because the urethra may be colonized.

Treatment of bacterial vaginosis has rested mainly with the use of metronidazole similar to the regimens used for the treatment of trichomoniasis. However, I believe that this agent should be used only for the first episode. If the patient fails to respond, consideration should be given to the possibility that the infection may be in the early stages of development with the flora dominated by *G. vaginalis*, as discussed earlier. Alternative therapy consists of the use of metronidazole gel or clindamycin cream. Oral clindamycin, 300 mg twice a day for 7–10 days, has been shown to be effective.[41] Other agents that can be used are amoxicillin/clavulanic acid, 500 mg given orally 3 times daily for 7–10 days. Ampicillin and amoxicillin have been shown to be only 30–45% effective in the treatment of bacterial vaginosis.[42] This observation is compatible with the hypothesis that this disease may well be a spectrum of microbiological change that begins with a decrease in lactobacilli, a rise in pH, and an increase in *G. vaginalis* growth. Treatment at this stage would most likely be effective since *G. vaginalis* is sensitive to these agents but resistant to metronidazole. Once the condition has progressed and the anaerobes have become dominant, the narrow-spectrum penicillins become ineffective.

### SUMMARY

Vaginitis due to "infection" can be defined as a disturbance in the microecosystem in which the endogenous microflora is prevented from maintaining its equilibrium. Commensal organisms exist in a synergistic state with one another and exert an antagonistic effect on potential pathogens. This disruption of the dynamic equilibrium of the healthy vagina may have significant sequelae. These patients may develop significant symptoms that prevent them from leading normal lives, often precluding them from having sexual relations because of odor or pain, and strain their relationships as a result. In some cases, these patients become "pelvic cripples." Another concern is the potential for developing serious conditions such as premature rupture of amniotic membranes, premature labor and delivery, or low birth weight when vaginitis is associated with pregnancy. Finally, vaginitis may predispose a patient undergoing pelvic or vaginal surgery to significant postoperative infection. For these reasons, the patient with vaginitis must always be taken seriously, the condition must never be written off as a mild insignificant condition, and an accurate diagnosis must always be established.

### REFERENCES

1. Paavonen J, Stamm WE: Lower genital tract infections in women. In Hansfield HH (ed): Sexually Transmitted Diseases. Infectious Disease Clinics of North America. Philadelphia: W.B. Saunders, pp 179–198, 1987.
2. Thomason JI, Gelbart SM, Broekhuizen FF: Office and clinical laboratory diagnosis of vulvovaginal infections: An overview. In Horowitz BJ, Mardh P-A (eds): Vaginitis and Vaginosis. New York: Wiley-Liss, pp 93–108, 1991.
3. Mardh P-A: Vaginal microbial ecology. In Horowitz BJ, Mardh P-A (eds): Vaginitis and Vaginosis. New York: Wiley-Liss, pp 1–9, 1991.
4. Dahiya RS, Speck ML: Hydrogen peroxide formation by lactobacilli and its effect on *Staphylococcus aureus*. J Dairy Sci 51:1568–1572, 1968.
5. Klebanoff JJ, Smith DC: Peroxidase-mediated antimicrobial activity of rat uterine fluid. Gynecol Invest 1:21–30, 1979.
6. Eschenbach DA, Davick PR, Williams BC: Prevalence of hydrogen peroxide producing *Lactobacillus* species in normal women and women with bacterial vaginosis. J Clin Microbiol 27:251–256, 1989.
7. Upreti GC, Hinsdill RD: Isolation and characterization of a bacteriocin from a homofermative lactobacillus. Antimicrob Agents Chemother 4:484–487, 1973.
8. Andersson RE, Daeschel MA, Hassan HN: Antibacterial activity of plantaricin SIK-83, a bacteriocin produced by *Lactobacillus plantarum*. Biochemistry 70:381–390, 1988.
9. Barefoot SK, Klaenhammer TR: Detection and activity of lactacin B, a bacteriocin produced by *Lactobacillus acidophilus*. Appl Environ Microbiol 45:1808–1815, 1983.
10. Reid G, Cook RL, Bruce AW: Examination of strains of lactobacilli for properties that may influence bacterial interference in the urinary tract. J Urol 138:330–335, 1987.
11. Sobel JD, Myers P, Levison ME, Kaye D: *C. albicans* adherence to vaginal epithelial cells. J Infect Dis 143: 767–782, 1981.
12. Cox SM, Phillips LE, Mercer LJ, Stager CE, Waller S, Faro S: Lactobacillemia of amniotic fluid origin. Obstet Gynecol 68:134–135, 1986.
13. Gardner HL, Dukes CD: *Haemophilus vaginalis* vaginitis. Am J Obstet Gynecol 69:962–976, 1955.
14. Faro S, Phillips LE: Non-specific vaginitis or vaginitis of undetermined etiology. Int J Tissue Reactions IX:173–177, 1987.
15. Faro S: Persistent vaginitis and vaginosis. In Horowitz BJ, Mardh P-A (eds): Vaginitis and Vaginosis. New York: Wiley-Liss, pp 237–245, 1991.
16. Sobel JD: Epidemiology and pathogenesis of recurrent vulvovaginal candidiasis. Am J Obstet Gynecol 152:924–935, 1985.
17. Friedrich EG Jr: Vulvar vestibulitis syndrome. J Reprod Med 32:110–114, 1987.
18. Pyka RE, Wilkinson EJ, Friedrich EG Jr, Croler BP: The histopathology of vulvar vestibulitis syndrome. Int J Gynecol Pathol 7:249–257, 1988.
19. Turner MLC, Marinoff SC: Association of human papilloma virus with vulvodynia and the vulvar vestibular syndrome. J Reprod Med 33:533–537, 1988.
20. Friedrich EG Jr: Therapeutic studies on vulvar vestibulitis. J Reprod Med 33:514–518, 1988.
21. Woodruff D, Friedrich EG Jr: The vestibule. Clin Obstet Gynecol 28:134–141, 1985.
22. Lossick JG: Treatment of *Trichomonas vaginalis* infections. Rev Infect Dis 4:S-801–818, 1982.
23. Hager WD, Brown ST, Klaus SJ, et al.: Metronidazole in vaginal trichomoniasis. Seven day vs. single-dose regimes. JAMA 244:1219–1220, 1980.
24. O'Reilly RA: Stereospecific interaction of warfarin and metronidazole. Fed Proc 34:259, 1975.

25. Roe JFC: A critical appraisal of the toxicology of metronidazole. In Phillips I, Collier J (eds): Metronidazole. London: Academic Press, pp 215–222, 1979.
26. Beard CM, Noller KL, O'Fallon WM, Kurkland LT, Dockerty MB: Lack of evidence for cancer due to use of metronidazole. N Engl J Med 301:519–522, 1979.
27. Friedman GD: Cancer after metronidazole (letter). N Engl J Med 302:519, 1980.
28. Peterson WF, Stauch JE, Ryder CO: Metronidazole in pregnancy. Am J Obstet Gynecol 94:343–349, 1966.
29. Erickson SH, Oppenheim GL, Smith GH: Metronidazole in breast milk. Obstet Gynecol 57:48–50, 1981.
30. Schnell JD: The incidence of vaginal *Candida* and *Trichomonas* infections and treatment of *Trichomonas vaginalis* with clotrimazole. Postgrad Med 50:79–81, 1974.
31. Pereyra AJ, Lansing JD: Urogenital trichomoniasis. Treatment with metronidazole in 2,002 incarcerated women. Obstet Gynecol 24:499–508, 1964.
32. Meingassner JG, Turner J: Strains of *Trichomonas vaginalis* resistant to metronidazole and other 5-nitro-imidazoles. Antimicrob Agents Chemother 15:254–257, 1979.
33. Lossik JG, Muller M, Gorrell TE: In vitro drug susceptibility and doses of metronidazole required for cure in cases of refractory vaginal trichomoniasis. J Infect Dis 153:948–955, 1986.
34. Muller M, Lossick JG, Gorrell TE: In vitro susceptibility of *Trichomonas vaginalis* to metronidazole and treatment outcome in vaginal trichomoniasis. Sex Transm Dis 15:17–24, 1988.
35. Frytak S, Mortel CG, Childs OS, Albers JW: Neurologic toxicity associated with high dose metronidazole. Ann Intern Med 88:361–362, 1978.
36. Redondo-Lopez V, Lynch M, Schmitt C: *Torulopsis glabrata* vaginitis: Clinical aspects and susceptibility to antifungal agents. Obstet Gynecol 76:651–655, 1990.
37. Cook CW: Vulvovaginal candidiasis (letter). Obstet Gynecol 48:631, 1976.
38. Miles MR, Olsen I, Rogers A: Recurrent vaginal candidiasis: Importance of an intestinal reservoir. JAMA 238: 1836–1837, 1977.
39. Nystatin Multicenter Study Group: Therapy of candidal vaginitis. The effect of eliminating intestinal *Candida*. Am J Obstet Gynecol 155:651–655, 1986.
40. Horowitz BJ, Edelstein SW, Lippman L: Sexual transmission of *Candida*. Obstet Gynecol 69:883–886, 1987.
41. Greaves W, Chungafung J, Morris B, Haile A, Townsend JF: Clindamycin versus metronidazole in the treatment of bacterial vaginosis. Obstet Gynecol 72:799–802, 1988.
42. Lee L, Schmale JD: Ampicillin therapy for *Corynebacterium vaginalae (Haemophilus vaginalis)* vaginitis. Am J Obstet Gynecol 115:786–788, 1973.
